# Site-directed mutagenesis in *Arabidopsis thaliana* using dividing tissue-targeted RGEN of the CRISPR/Cas system to generate heritable null alleles

**DOI:** 10.1007/s00425-014-2180-5

**Published:** 2014-10-01

**Authors:** Youbong Hyun, Jungeun Kim, Seung Woo Cho, Yeonhee Choi, Jin-Soo Kim, George Coupland

**Affiliations:** 1 Max Planck Institute for Plant Breeding Research, 50829 Cologne, Germany; 2 National Creative Research Initiatives Center for Genome Engineering, Seoul National University, 151-747 Seoul, Korea; 3 Department of Chemistry, Seoul National University, 151-747 Seoul, Korea; 4 Department of Biological Sciences, Seoul National University, 151-747 Seoul, Korea

**Keywords:** RGEN, CRISPR/Cas system, Site-directed mutagenesis, *ICU2*

## Abstract

*****Main conclusion***:**

**Dividing tissue-targeted site-directed mutagenesis using RGEN of CRISPR/Cas system produces heritable mutations in** ***Arabidopsis thaliana.***

**Abstract:**

Site-directed genome engineering in higher plants has great potential for basic research and molecular breeding. Here, we describe a method for site-directed mutagenesis of the *Arabidopsis* nuclear genome that efficiently generates heritable mutations using the RNA-guided endonuclease (RGEN) derived from bacterial clustered regularly interspaced short palindromic repeats (CRISPR)-Cas9 (CRISPR associated) protein system. To induce mutagenesis in proliferating tissues during embryogenesis and throughout the plant life cycle, the *single guide RNA* (*sgRNA*) and Cas9 DNA endonuclease were expressed from the *U6 snRNA* and *INCURVATA2* promoters, respectively. After *Agrobacterium*-mediated introduction of T-DNAs encoding RGENs that targets *FLOWERING LOCUS T* (*FT*) and *SQUAMOSA PROMOTER BINDING PROTEIN*-*LIKE 4* genes, somatic mutagenesis at the targeted loci was observed in T1 transformants. In the results of FT-RGEN, T1 plants often showed late flowering indicative of the presence of large somatic sectors in which the *FT* gene is mutated on both chromosomes. DNA sequencing analysis estimated that about 90 % of independent chromosomal DNA fragments carried mutations in the analyzed tissue of a T1 plant showing late flowering. The most frequently detected somatic polymorphism showed a high rate of inheritance in T2 plants, and inheritance of less frequent polymorphisms was also observed. As a result, late-flowering plants homozygous for novel, heritable null alleles of *FT* including a 1 bp insertion or short deletions were recovered in the following T2 and T3 generations. Our results demonstrate that dividing tissue-targeted mutagenesis using RGEN provides an efficient heritable genome engineering method in *A. thaliana*.

**Electronic supplementary material:**

The online version of this article (doi:10.1007/s00425-014-2180-5) contains supplementary material, which is available to authorized users.

## Introduction

Precise and efficient genome targeting technologies in higher plants create great opportunities for basic research and molecular breeding. Although diverse targeted genome engineering techniques, such as zinc finger nuclease (ZFN) and transcription activator-like effectors nuclease (TALEN), have been applied in various higher organisms (Gaj et al. 2013), there remains a need for new technologies that are efficient, robust, and easy to engineer.

Recent advances in the study of prokaryotic adaptive immune system, type II clustered regularly interspaced short palindromic repeats (CRISPR) and CRISPR-associated endonuclease (Cas) provide a tool for new approaches in targeted genome engineering (Cho et al. 2013a; Cong et al. 2013; Hwang et al. 2013a; Mali et al. 2013). Type II CRISPR/Cas systems are widespread in bacteria and rely on targeted foreign DNA cleavage by RNA-guided endonuclease (RGEN) activity (Bhaya et al. 2011). The CRISPR locus consists of four genes, including the Cas9 nuclease, and two noncoding RNAs; trans-activating crRNA (tracrRNA) and a precursor crRNA (pre-crRNA) containing an array of nuclease guide sequences that were previously captured from potential pathogens. Transcribed pre-crRNAs are further processed by unknown mechanisms and the mature crRNA forms the RNA–protein complex with tracrRNA and Cas9 endonuclease. Twenty nucleotide guide sequences in crRNA participate in guiding the crRNA–tracrRNA–Cas9 complex to complementary DNA sequences of the target site. All of the target sites of the CRISPR/Cas system are composed of protospacer motifs that anneal with the guide sequence of crRNA and an adjacent 3′ protospacer adjacent motif (PAM) comprising NGG nucleotide sequences. PAM is responsible for target site verification by Cas9, and then Cas9 induces DNA cleavage after recognition of PAM when the crRNA–tracrRNA–Cas9 complex binds to the target sites (Sternberg et al. 2014). Chimeric single guide RNAs (sgRNA) have been constructed by fusion of the tracrRNA and crRNA in a single transcriptional unit (Jinek et al. 2012). Additionally, engineering of guide sequences complementary to selected genomic loci have revealed that the chimeric sgRNA can efficiently guide the Cas9 enzyme to potentially any genomic region of interest as long as the site homologous to the guide RNA is followed by an NGG nucleotide sequence motif (Jinek et al. 2012).

Many site-directed mutagenesis technologies depend on the introduction of errors caused by DNA repair systems after the induction of DNA double strand breaks (DSB) at specific sites. DSBs are repaired by non-homologous end joining (NHEJ) in diverse organisms including higher plants (Lieber 2010), and during this process nucleotide exchange, insertion or deletion of nucleotides occurs at low frequencies. Consistently, introduction of RGEN to cultured human cell populations induced mutations only in a portion of cells (Cho et al. 2013a; Cong et al. 2013; Mali et al. 2013). In addition, transfection of RGEN components in single-cell fertilized eggs of zebrafish generated chimeric individual organisms harboring mutant sectors (Hwang et al. 2013b). In higher plants, RGEN-mediated site-directed mutagenesis has been used in tobacco leaves and callus, rice callus culture, and protoplasts made from tobacco and *Arabidopsis* mesophyll cells (Li et al. 2013; Nekrasov et al. 2013; Shan et al. 2013). Similar to the animal studies, all of these approaches induced mutations at targeted sites in a portion of cells. In the cases of tobacco and rice, mutant plants were regenerated from RGEN-applied callus culture after selection of mutated plantlets. However, transformation of the widely used model species *Arabidopsis thaliana* is based on infiltration of *Agrobacterium tumefaciens* into developing inflorescences and selection of transformed progeny without a requirement for callus culture or plantlet regeneration (Clough and Bent 1998). A current challenge is to develop methods that combine efficient mutagenesis using RGEN in *Arabidopsis* with tissue-culture independent transformation methods. Such a method would allow targeting of loci for which T-DNA insertion alleles are not present in current populations (Sessions et al. 2002), and would enable the generation of stable null alleles at loci for which T-DNA insertions in coding sequences are not available. Previously, ZFN-mediated mutagenesis was applied to the *ABA*-*INSENSITIVE4* (*ABI4*) gene in *Arabidopsis* after transgene integration in the nuclear genome (Osakabe et al. 2010). Transient induction of ZFN using the heat shock inducible promoter for ZFN expression resulted in ~3 % of mutated chromosomal DNA at the *ABI4* locus in analyzed leaf tissues. The authors also isolated homozygous *abi4* loss of function mutants that exhibited the expected mutant phenotypes in the following generations. Similar approaches were recently performed using TALENs and RGENs in transgenic *Arabidopsis* plants in which the mutagenic components were expressed under the *CaMV 35S* promoter, and the identification of somatic mutations and mutant progeny were presented (Christian et al. 2013; Feng et al. 2013, 2014), suggesting a potential way to obtain heritable site-directed mutagenesis through reproduction of transgenic plants that contain the mutagenic components.

In this study, we introduced the RGEN technology of the CRISPR/Cas system in *A. thaliana* to establish a heritable site-directed mutagenesis system. To increase the transmission rate of mutant polymorphisms to the progeny, we targeted the mutagenic activity to the proliferating tissues in plants using a dividing tissue specific promoter to express Cas9. As a result, we observed highly efficient somatic mutagenesis at genomic targets in *Arabidopsis* transgenic plants, and the mutant polymorphisms were readily transmitted to the following generations resulting in a high frequency of progeny exhibiting the expected strong mutant phenotypes. The spectrum of somatic and heritable mutant alleles recovered is described. Taken together, our results provide an efficient and powerful tool for the isolation of stable, transmissible alleles by site-directed mutagenesis in *A. thaliana* and related species that can be transformed independently of tissue culture.

## Materials and methods

### Plant materials, growing conditions, histochemical GUS staining, and microscopy

All plants used in this study are in *Arabidopsis thaliana* Col-0 background. Plants were grown in long-day (16 h light/8 h dark) photoperiodic conditions under cool white fluorescence light (100 μmol/m^2^/s) at 22 °C with 60 % relative humidity. GUS activity in developing seeds was analyzed by incubation of seeds in 50 mM NaPO_4_ (pH 7.0), 1 mM X-Gluc, 1 mM K_3_Fe(CN)_6_, 1 mM K_4_Fe(CN)_6_, 10 mM EDTA, and 0.2 % Triton X-100 at 37 °C for 8–10 h. After staining, the tissues were cleared by incubation in 70 % ethanol for several hours. The stained tissues were photographed using an Axio Imager A1 microscope with an AxioCam HRc camera (Carl Zeiss).

### Construction of RGEN transgenic plants

To construct a binary vector for RGEN, protein coding sequence of Cas9 was cloned from p3s-Cas9hc plasmid (Cho et al. 2013a) to binary vector pGreen0229 (Hellens et al. 2000) through restriction enzyme-mediated excision and ligation processes. Identical region of *INCURVATA2* (*ICU2*) promoter that was used in *ICU2p*::*GUS* construction was amplified by PCR using Phusion High-Fidelity DNA polymerase (Thermo) from Col-0 genomic DNA. The amplified DNA was directly cloned upstream of Cas9 protein coding sequence and the clone with no nucleotide sequence error was isolated. This binary vector called pYB196 was subsequently used to insert different *U6p*::*sgRNA* constructs, and the sequence of the pYB196 vector is provided (NCBI GenBank accession number KJ816368). To generate *U6p*::*sgRNA*, an overlapping PCR technique was applied. The principle of the technique is described in the “Results” section and Fig. 2. Overlapping PCR was performed using two amplified products of *U6p* and *sgRNA* PCR fragments as templates after purification. To provide equal numbers of DNA molecules of each fragment in the overlapping PCR, 18 ng of *U6p* and 11 ng of *sgRNA* PCR products that represent 100 fmol of DNA molecules were used after quantification. The amplified products were directly cloned into binary vector pYB196. Sequences of primers used in these processes are presented in Supplemental Table S5. To obtain RGEN transgenic plants, clones without sequence error were transformed to *Arabidopsis* Col-0 plants through *Agrobacterium*-mediated floral dipping (Clough and Bent 1998) and then T1 transgenic plants were identified by Basta selection.

### T7E1 assay for detecting mutation

To detect mutation on targeted genomic loci, the genomic DNA was extracted from cauline leaves at the position below the first open flower of T1 transformants. The harvested leaf tissue was ground using blue pestle within 500 μl 2× CTAB buffer (100 mM Tris–Cl pH 8.0, 1.4 M NaCl, 20 mM EDTA, 2 % CTAB). The same volume of chloroform was mixed with the ground sample solution by vortex. The mixture was incubated at 65 °C for 15 min and then was centrifuged at 12,000 rpm at 10 min. The resulted supernatant was transferred to new tube containing equal volume of isopropanol. DNA was precipitated at −20 °C for 30 min after gentle mixture of the solution and was collected by centrifugation at 12,000 rpm at 15 min. The produced pellet of DNA was dissolved in DNase-free water for further study. The target genomic region was amplified by nested PCR and the amplified PCR products were denatured and reannealed (95 °C; 2 min, damp to 85 °C; 2 °C/s, damp to 25 °C; 0.1 °C/s). The reannealed PCR products were digested with T7E1 nuclease (New England Biolabs) which specifically cleaves DNA with mismatches at 37 °C for 20 min. Digested PCR products were separated and detected on agarose gel containing ethidium bromide.

### Sequencing analysis for characterization of polymorphisms

To characterize the polymorphisms at target sites, genomic region which includes the target site of each RGEN was amplified by PCR using Phusion High-Fidelity DNA polymerase (Thermo). The amplified PCR products were cloned to pGEM-T easy vector (Promega) after A-tailing of PCR products (72 °C; 30 min with ordinary Taq polymerase). After *E. coli* transformation, the amplified region in each clone was sequenced.

## Results

### *ICU2p*::*Cas9/U6p*::*sgRNA* T-DNA cassette for RGENs in plants

**Fig. 1:**
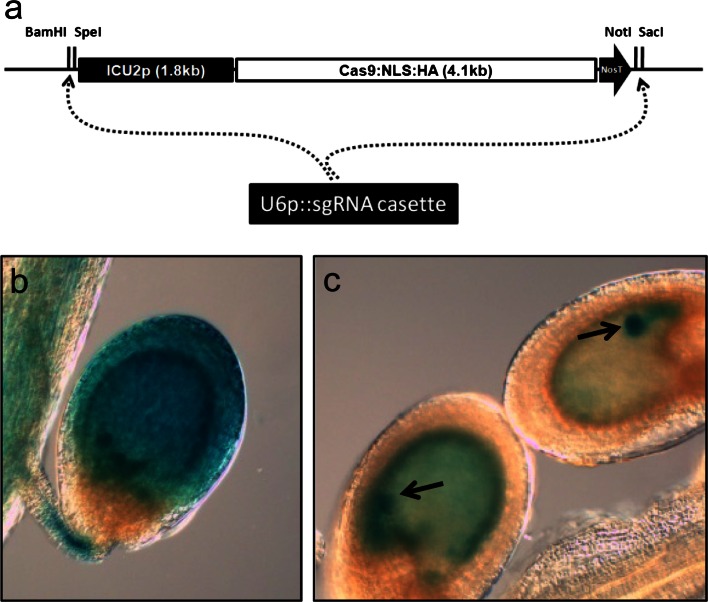
RGEN for heritable site-directed mutagenesis in *Arabidopsis*. **a** Schematic diagram of the T-DNA of the RGEN binary vector used in this study. *U6p*::*sgRNA* genes can be cloned into the pYB196 binary vector that carries *ICU2p*::*Cas9* using four different restriction enzyme sites. Potentially more than one *U6p*::*sgRNA* could be expressed from one T-DNA for multiplex mutagenesis. GUS staining of *ICU2p*::*GUS* plants after self fertilization (**b**) and of F1 seeds made by fertilizing wild-type Col with pollen from *ICU2p*::*GUS* plants (**c**). As *ICU2p*::*GUS* transgene was introduced paternally in **c**, only the embryo and endosperm of the F1 seeds possess the transgene. *Arrows* indicate embryo in developing seeds

All of the aerial organs in higher plants are generated from meristematic tissues in shoot apices. Site-directed mutagenesis techniques, including RGEN, induce mutations in only a proportion of cells that then create somatic sectors through cell division (Gaj et al. 2013). By expressing Cas9 and *sgRNA* in shoot apical meristems RGEN activity is expected to produce somatic mutant sectors, some of which will encompass floral primordia and lead to the transmission of mutations through the gametes. To express Cas9 endonuclease in meristematic regions, we fused the promoter of *INCURVATA2* (*ICU2*) to the Cas9 protein coding DNA which was used in RGEN of human cells (Fig. 1a) (Cho et al. 2013a). *ICU2* encodes a catalytic subunit of DNA polymerase α in *Arabidopsis*. The *ICU2* promoter is highly active in cells that frequently divide, including those in meristems, proliferating primordia, the vegetative shoot, inflorescences and flowers (Hyun et al. 2013), supporting the characterized function of the gene. To examine if the promoter of *ICU2* is also active in developing embryos, β-Glucuronidase (GUS) activity was analyzed in transgenic *A. thaliana* plants carrying *ICU2p*::*GUS* (Fig. 1b, c). In the seeds of *ICU2p*::*GUS* plants, GUS activity in the seed coat prevented observation of the signal in the developing embryo (Fig. 1b). Therefore, to exclude transgene expression in maternal tissues of seeds, we pollinated wild-type with pollen of *ICU2p*::*GUS* plants. In the resulting hybrids, strong GUS expression was detected in the developing embryo and endosperm, indicating that the *ICU2* promoter is active in these tissues (Fig. 1c). Together, these *ICU2p*::*GUS* staining results suggest that *ICU2p*::*Cas9* will express the DNA endonuclease Cas9 in proliferating tissues of *A. thaliana* from embryogenesis and throughout somatic development.

Chimeric *sgRNA* guides Cas9 protein to target genomic sites by RNA–protein complex formation. To optimize its expression, we used the promoter of *A. thaliana* U6 snRNA gene (Supplemental Fig. S1a). *U6 snRNA* is transcribed by RNA polymerase (III), and the polymerase recognizes promoters with a simple structure and TTTTT motif as a transcriptional terminator (Li et al. 2007) (Supplemental Fig. S1a) that is beneficial in expressing short RNA molecules with the intended sequence context. The *U6 snRNA* promoter has been used in induction of short-hairpin RNA for RNAi in animal cells (Makinen et al. 2006) and for expression of *sgRNA* for RGEN techniques in several organisms (Cho et al. 2013a; Li et al. 2013; Nekrasov et al. 2013; Shan et al. 2013). Among the *U6 snRNA* family genes in *A. thaliana*, the promoter of *U6*-*26 snRNA* gene was employed in this study as it was shown that *U6*-*26 snRNA* is expressed most strongly in all tissues analyzed, including shoot apices (Li et al. 2007). In the *A. thaliana* genome, *U6 snRNA* genes are closely located to several protein coding genes that are transcribed by RNA polymerase (II) (Supplemental Fig. S2). Thus, this promoter can be utilized in the context of a T-DNA containing genes expressed by RNA polymerase (II).

**Fig. 2:**
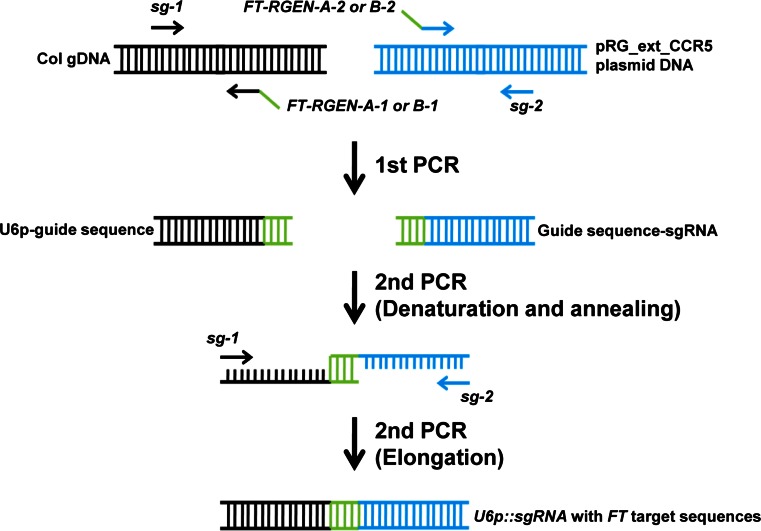
Cloning of *U6p*::*sgRNA* cassette by overlapping PCR. To construct the *U6p*::*sgRNA* cassette containing a target specific guide sequence, the guide sequence (*green* *color*) is added at the 3′ and 5′ ends of the *U6* promoter (*black* *color*) and sgRNA backbone (*blue* *color*) during PCR amplification. In a second round of PCR, those two PCR products are annealed together by the guide sequence. The target specific *U6p*::*sgRNA* cassette is then amplified in a second overlapping PCR. *Colored ladders* present double stranded DNA molecules. Primers used in the cloning of FT-RGENs are marked with the *arrows* and *letters* in *italics*

To facilitate the use of the RGEN system in *A. thaliana*, a binary vector was constructed based on pGREEN0229 (Hellens et al. 2000). This binary vector contains *ICU2p*::*Cas9* and four adjacent unique restriction sites into which *U6p*::*sgRNA* genes can be inserted (Fig. 1a). The sequence of this vector, called pYB196, is available at the GenBank in NCBI (accession number KJ816368). *U6p*::*sgRNA* was then made by overlapping PCR (Fig. 2). The promoter of *U6*-*26 snRNA* was amplified from Col-0 genome by PCR using the promoter specific primers. Target specific 20 bp guide sequence of *sgRNA* was attached at the 3′ end of *U6p* PCR products by primer-mediated extension. *SgRNA* backbone fragment which contains the transcriptional terminator motif of RNA pol (III) was amplified from pRG_ext_CCR5 plasmid (Cho et al. 2013a). The guide sequence was added at the 5′ end of *sgRNA* backbone. These two PCR products were then used in the second round of overlapping PCR as templates. The guide sequence attached at the 3′ and 5′ ends of *U6p* and *sgRNA* backbone, respectively, allowed annealing of the two PCR products. The overlapping PCR therefore produced the fused *U6p*::*sgRNA* cassette carrying the target specific guide sequence (Supplemental Fig. S1b). By adding restriction enzyme sites in the primers used in the second PCR, the amplified *U6p*::*sgRNA* cassette could be directly cloned in the pYB196 binary vector. In principle, four distinct *U6p*::*sgRNAs* could be expressed from the same vector. Sequences of primers used in the cloning of *U6p*::*sgRNA* cassette are presented in Supplemental Table S5. In this way, a binary vector harboring *ICU2p*::*Cas9* and *U6p*::*sgRNA* in the same T-DNA (Fig. 1a) is constructed to facilitate establishment of *Arabidopsis* transformants that stably express both factors after a single floral dipping procedure.

### Targeted mutagenesis of *FT* and *SPL4* loci using RGENs

**Fig. 3:**
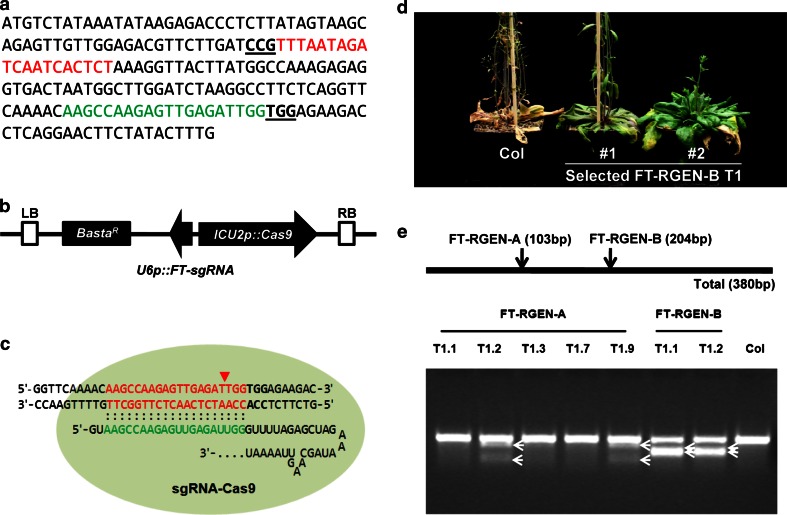
Site-directed mutagenesis at *FT* using FT-RGENs. **a** The nucleotide sequence of the first exon of *FT*. Two target sites for FT-RGEN-A and FT-RGEN-B are presented with sequences in *red* and *green*, respectively. The PAM sequences are marked in *bold underlined letters*. **b** Schematic structure of FT-RGEN T-DNA. *U6p*::*sgRNA* that possesses a guide sequence for *FT* targeting was cloned upstream of *ICU2p*::*Cas9* in reverse orientation (*black arrow boxes*). *Basta* ^*R*^ gene is the selectable marker for identification of transgenic plants (*black box*). Left border (*LB*) and right border (*RB*) of T-DNA are presented with *white boxes*. **c** Schematic structure of RNA-guided Cas9 at FT-RGEN-B target site. Double-stranded genomic sequence of *FT* and a part of the sequence of chimeric sgRNA for *FT* targeting are presented. Target sequence of FT-RGEN-B and the guide sequence in sgRNA are presented in *red* and *green characters*, respectively. Expected DNA cleavage site by RGEN is marked with the *red triangle*. PAM sequence adjacent to the 3′ end of the target site is presented in *bold characters*. **d** Recapitulation of the *ft* mutant phenotype in T1 transgenic plants of FT-RGEN-B. Two independent T1 transgenic plants showing late-flowering phenotypes are presented. **e** Polymorphism test using the T7E1 assay. Schematic structure of the PCR amplicon for the T7E1 assay is presented in the *top panel*. *Black arrows* indicate the positions of two RGEN target sites that would be digested by T7E1 enzyme if induced mutations are present. T7E1-mediated cleaved products are marked with *white arrows* in the *bottom panel*

To examine whether our RGEN system induces site-directed mutagenesis in *A. thaliana*, we first chose two target sites in the first exon of *FLOWERING LOCUS T* (*FT*) (Fig. 3a), one of the major floral integrators (Andres and Coupland 2012; Kardailsky et al. 1999; Kobayashi et al. 1999), and constructed RGEN T-DNAs designed to express chimeric *sgRNAs* with guide sequences against the target sites (Fig. 3b, c). After *Agrobacterium*-mediated floral infiltration, 12 and 11 T1 transgenic plants were isolated that carry the T-DNA of FT-RGEN-A or FT-RGEN-B in their genome, respectively. Interestingly, 9 T1 plants carrying FT-RGEN-B exhibited delayed flowering of differing severity (Fig. 3d; Supplemental Table S1), whereas the T1 plants carrying FT-RGEN-A flowered at a similar time to wild-type. Considering that the loss of *FT* function in *A. thaliana* results in a late-flowering phenotype (Kardailsky et al. 1999; Kobayashi et al. 1999; Koornneef et al. 1991), the delayed flowering of FT-RGEN-B T1 plants strongly suggests the induction of site-directed mutagenesis at the target site.

To test whether there are polymorphisms in these plants induced by RGENs, T7 endonuclease 1 (T7E1) digestion analysis was employed. The T7E1 DNA endonuclease specifically recognizes and cleaves the mismatches in double-stranded DNA molecules. Genomic DNA of Col and 7 of the FT-RGEN T1 transformants were extracted from cauline leaves. In each case, the cauline leaf at the position below the first open flower was used, and the RGEN target genomic region was amplified by PCR. The PCR products were denatured and reannealed and then incubated with T7E1 enzyme. Digested DNA products were detected from several of the FT-RGEN transgenic plants, but not from wild-type plants (Fig. 3e). Additionally, the digested products for each FT-RGEN plant exhibited the expected size separation after DNA electrophoresis. This analysis suggested the presence of RGEN induced mutations at both target sites within the *FT* genomic locus. PCR products from genomic DNA of FT-RGEN-B T1 plants produced more abundant digested products in comparison to those of FT-RGEN-A T1 plants. This result suggests that FT-RGEN-B T1 plants contained more mutant sectors in the analyzed tissues than FT-RGEN-A T1 plants, and this conclusion is in agreement with the differences in phenotypic severity between transformants carrying FT-RGEN-A and FT-RGEN-B.

**Fig. 4:**
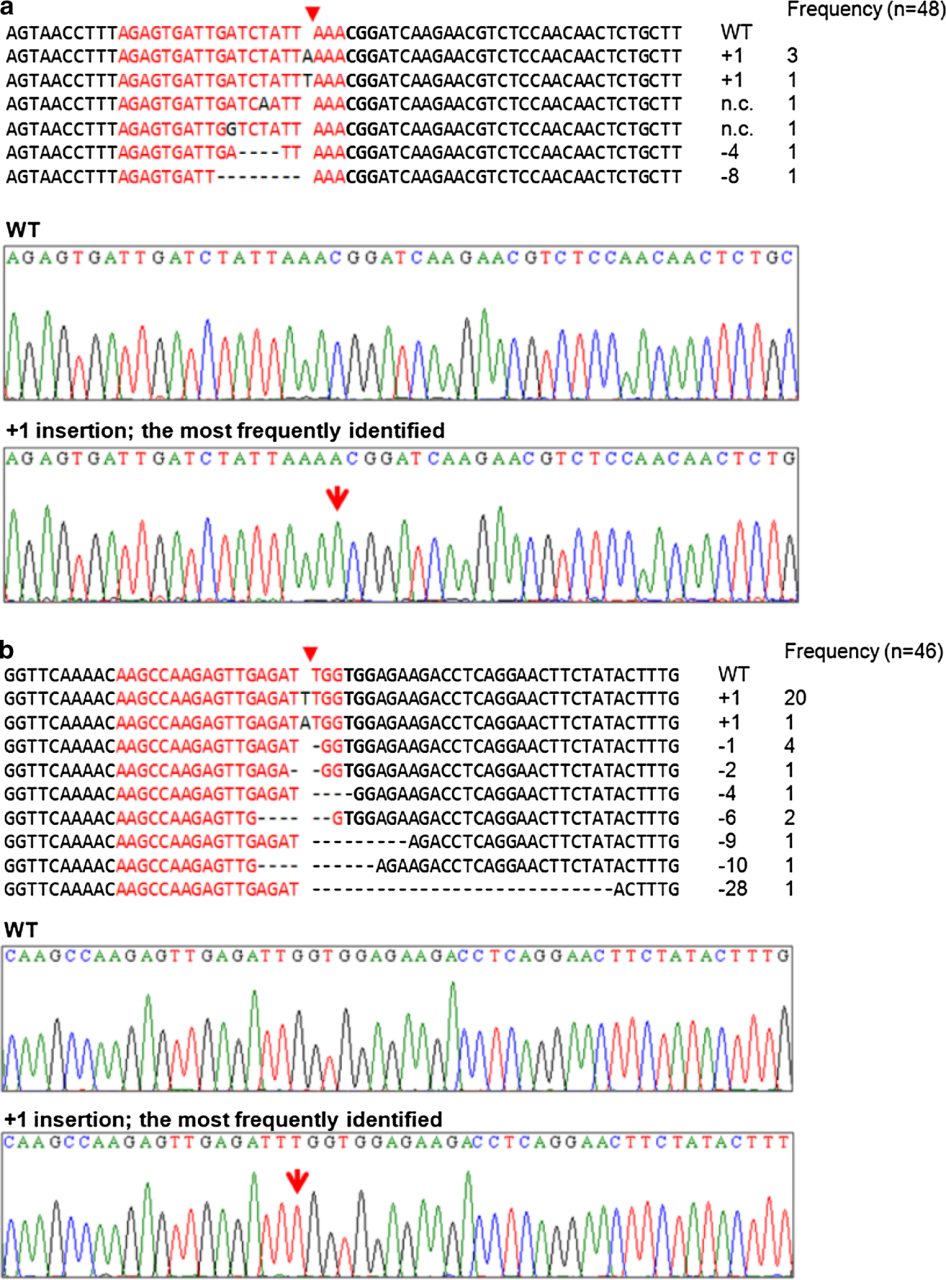
FT-RGEN-induced polymorphisms. Detected polymorphisms in FT-RGEN-A T1.2 (**a**) and FT-RGEN-B T1.2 (**b**) plants. Representative polymorphisms are presented in the *top panel*. The predicted DNA cleavage site is marked with a *red triangle*. Nucleotide numbers of insertions or deletions and the frequency of each polymorphism are presented to the *right* of each polymorphism. Sequence peaks of wild-type and the most frequent polymorphism are presented at the *bottom* of the *panel*. Sequence peak of inserted nucleotide is marked with a *red arrow*

The RGEN target sites were then analyzed in two T1 transformants, FT-RGEN-A T1.2 and FT-RGEN-B T1.2, by sequencing of individual clones of the amplified PCR products. As a result, somatic mutations that included nucleotide deletions, insertions and exchanges were identified at the target sites of FT-RGEN-A and FT-RGEN-B (Fig. 4a, b). Consistent with the T7E1 assay results (Fig. 3e), the FT-RGEN-A T1.2 plant showed a relatively lower rate of mutagenic efficiency than the FT-RGEN-B T1.2 plant (Table 1). Remarkably, one type of polymorphism bearing a single T insertion at the cleavage site of Cas9 was found very frequently in the FT-RGEN-B T1.2 plant (20 of 46 analyzed DNA) (Fig. 4b). The high frequency with which this polymorphism was recovered suggests that the mutation occurred prior to cauline leaf development and became incorporated into a sector that included the founder cells of the analyzed cauline leaf.

**Table 1:** Summary of FT-RGEN- and SPL4-RGEN-mediated site-directed mutagenesis in *A. thaliana*

Transgenic line	Ratio of mutated DNA	Numbers of polymorphisms			
		Total	Insertion	Deletion	Nucleotide exchange
FT-RGEN-A T1.2	18.75 % (9/48)	7	3	2	2
FT-RGEN-B T1.2	84.78 % (39/46)	15	4	11	0
SPL4-RGEN T1.8	10.00 % (4/40)	3	0	3	0

*SQUAMOSA PROMOTER BINDING PROTEIN*-*LIKE 4* (*SPL4*) genomic locus was additionally challenged to examine the mutagenic activity of our RGEN system. The *U6p*::*sgRNA* cassette that targets *SPL4* was inserted into pYB196 binary vector in the reverse orientation to that of *U6p*::*sgRNA* in FT-RGENs (Supplemental Fig. S3a). Among 40 analyzed individual target genomic site DNA clones derived from T1 transgenic plant, 4 showed the induced mutations in *SPL4* (Table 1 and Supplemental Fig. S3b). Taken together these results demonstrate that the *ICU2p*::*Cas9/U6p*::*sgRNA RGEN* system efficiently induces mutations at different loci that become efficiently established in large somatic sectors as the plant grows.

To examine the specificity of RGENs in *A. thaliana*, potential off-target sites for each FT-RGEN were searched for using Cas-OFFinder (http://www.rgenome.net) (Supplemental Table S2). All of the identified potential off-targets of FT-RGENs possess 3–4 bp mismatches against the guide sequences in *sgRNAs* and are followed by a NGG motif. Off-target sites that possess 3 bp mismatches were analyzed in FT-RGEN-A T1.2 and FT-RGEN-B T1.2 by sequencing in the same way as used to identify somatic mutations at the primary target site (Table 2). All of the analyzed clones from the off-target sites showed wild-type sequences. Additionally, we also examined the mutagenic activity of FT-RGENs on the guide sequence in the *U6p*::*sgRNA* transgenes. Although the guide sequence in the *U6p*::*sgRNA* gene provides the complementary binding site for *sgRNA* expressed from the transgene, the guide sequence in the *sgRNA* lacks the NGG motif that is crucial for target site validation of Cas9 (Sternberg et al. 2014). Consistent with the proposed role of the NGG motif, we detected no induced mutations in the examined clones of the guide sequence in the *U6p*::*sgRNA* transgenes (Table 2). Taken together, these analyses demonstrate that complementary binding of *sgRNA* at the target site followed by a NGG motif are essential for DNA cleavage induction by Cas9 in *A. thaliana* transformed plants.

**Table 2:** Sequence analysis of *sgRNA* transgenes and off-targets of FT-RGENs in T1 transformants

Transgenic line	Name of locus	Sequence^a^	Frequency of mutated DNA (total *n* = 48)
FT-RGEN-A T1.2	sgRNA	AGAGTGATTGATCTATTAAAGTT	0
	off-target-1	AGAG*c*GATTGA*a*CTAT*a*AAA**CGG**	0
	off-target-11	AGA*c*T*a*ATTGAT*a*TATTAAA**TGG**	0
FT-RGEN-B T1.2	sgRNA	AAGCCAAGAGTTGAGATTGGGTT	0
	off-target-12	AA*a*CCAA*t*AGT*g*GAGATTGG**AGG**	0

^a^Mismatched nucleotides and PAM motif in the analyzed off-targets are marked with italicized lower case and bold upper case letters, respectively

### Stable inheritance of induced mutations and establishment of homozygous mutants of *ft*

**Fig. 5:**
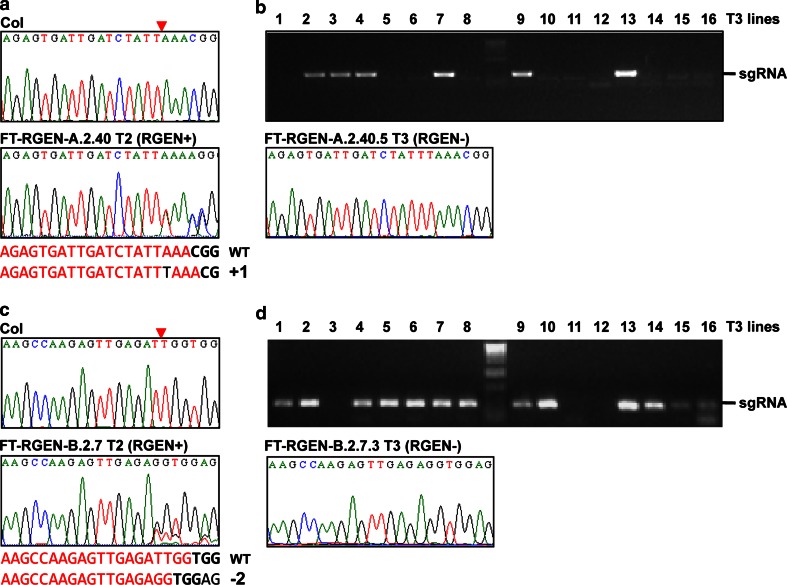
Inheritance of induced polymorphisms during reproduction in FT-RGENs. **a** Sequencing results of FT-RGEN-A.2.40 T2 plant. Sequence peaks of wild-type and the heterozygous candidate T2 are presented. DNA cleavage site by Cas9 is marked with a *red triangle*. Nucleotide sequences of wild-type and the mutated target sites are presented with *letters* in *red* and the single T insertion in the mutated target site is presented with the *black letter*. PAM motifs are also marked with *bold black letters*. **b** Independent segregation of the inherited mutant allele and the RGEN transgene in T3 progeny of FT-RGEN-A.2.40. Result of the genotyping PCR to detect FT-RGEN-A transgene in the independent T3 individuals is presented in the *top panel*. Primers specific for the sgRNA were used in the genotyping PCR. Sequence peaks of the homozygous (FT-RGEN-A.2.40.5) mutant T3 plant that did not inherit the RGEN transgene are shown in the *bottom panel*. **c** Sequencing results of FT-RGEN-B.2.7 T2 plant. Sequence peaks of wild-type and the heterozygous candidate T2 are presented. DNA cleavage site by Cas9 is marked with a *red triangle*. Nucleotide sequences of wild-type and the mutated target sites are presented with *letters* in *red*. PAM motifs are also marked with *bold black letters*. **d** Independent segregation of the inherited mutant allele and the RGEN transgene in T3 progeny of FT-RGEN-B.2.7. Result of the genotyping PCR to detect FT-RGEN-B transgene in the independent T3 individuals is presented in the *top panel*. Sequence peaks of the homozygous (FT-RGEN-B.2.7.3) mutant T3 plant that did not inherit the RGEN transgene are shown in the *bottom panel*

**Fig. 6:**
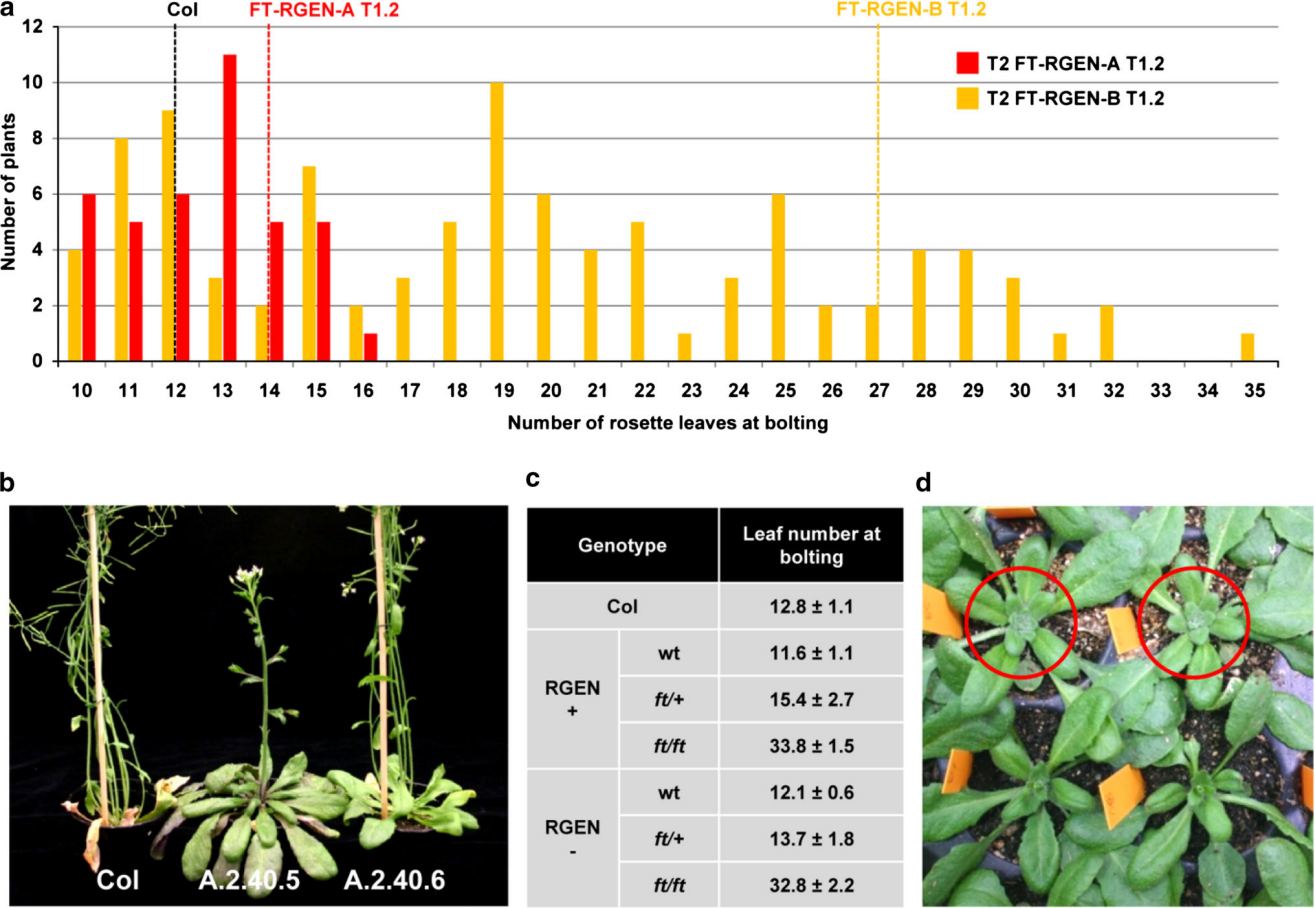
Flowering phenotypes of T2 and T3 progeny of FT-RGENs. **a** Distribution of flowering times among T2 individuals of FT-RGEN-A.2 and FT-RGEN-B.2. Average flowering times of wild-type Col, FT-RGEN-A T1, and FT-RGEN-B T1 mother plants are presented with *black*, *red* and *orange dashed lines*, respectively. **b** Picture of the isolated RGEN(−) ft homozyote (A.2.40.5) and heterozygote (A.2.40.6) in FT-RGEN-A.2.40 T3 progeny. **c** Average flowering times of T3 progeny of different genotypes derived from FT-RGEN-A.2.40. Flowering time of each genotype among the T3 progeny is illustrated using the average number of rosette leaves at bolting. **d** Picture of FT-RGEN-B T2 plants (marked with *red circles*) showing late-flowering mutant phenotype

To examine if the RGEN transgenic plants transmit the induced *ft* mutant alleles to their progeny, the genotypes of T2 progeny of the FT-RGEN-A.2 and FT-RGEN-B.2 T1 plants used to detect somatic mutations were examined. Genomic DNA was extracted from 2 to 3 leaves of 12-day old T2 seedlings for genotyping. The existence of the RGEN transgene was confirmed by genotyping PCR using the *sgRNA* specific primers and the genotypes of the target sites were analyzed by sequencing of target site PCR products. In 34 T2 progeny of FT-RGEN-A, one heterozygous *ft* mutant candidate was isolated (Fig. 5a), but no *ft* homozygous plants were recovered. Consistent with genotyping results, all of the T2 progeny of FT-RGEN-A exhibited similar flowering times to wild-type (Fig. 6a). The mutant allele found in the characterized *ft* heterozygous plant had been observed in the sequencing of the T1 mother plant (Fig. 4a). This result suggests that the mutant allele was inherited from the T1 plant, but because the FT-RGEN-A transgene was also present in this T2 plant it is also possible that this allele was induced independently in the T2. To further confirm the transmission of the polymorphism, T3 progeny of this T2 plant were genotyped for the FT-RGEN-A transgene and the mutant allele. In T3 progeny of the FT-RGEN-A.2.40 T2 *ft* heterozygous candidate, the mutant allele segregated independently from the FT-RGEN-A transgene (Fig. 5b; Supplemental Table S3), demonstrating that the mutant allele is heritable. Consistent with this genotyping result, the late-flowering phenotypes characteristic of *ft* were frequently observed in the FT-RGEN (–) T3 progeny (Fig. 6b, c; Supplemental Table S3). Of 61 FT-RGEN (–) T3 progeny isolated, 30 and 23 plants were found to be homozygous and heterozygous, respectively, for the *ft* mutant allele that had been identified both in T1 and T2 plants (Supplemental Table S3). As the ratio of homozygous and heterozygous mutant progeny is higher than the expected ratio of those from heterozygous mutant plants, more than half of the gametes of FT-RGEN-A.2.40 T2 plant were assumed to carry the mutation. This observation suggests that the mutant allele was inherited from T1 to T2 generation but that the FT-RGEN-A transgene induced the same type of mutant allele in the T2 plant by further rounds of mutagenesis (see “Discussion” section). In contrast to the inheritance of the induced mutation, FT-RGEN-A transgene exhibited a lower rate of transmission in the T3 progeny (29 RGEN (+) T3 plants of total 90 T3 progeny), suggesting that the transgene might reduce the reproductive success of gametes containing the RGEN in this transgenic lineage.

Among the T2 plants in the FT-RGEN-B lineage, several exhibited late-flowering phenotypes of varying severity (Fig. 6a, d). However, classical Mendelian segregation of late-flowering phenotypes in the T2 population was difficult to detect (Fig. 6a). This broad distribution in severity of the flowering defect could be due to the inheritance of *ft* mutant alleles differing in strength or to chimeric T2 plants caused by FT-RGEN-B inducing further rounds of mutagenesis in this generation. Of 23 T2 plants tested, one plant was identified as a *ft* heterozygous mutant that did not contain FT-RGEN-B (B.2.10 T2 plant in Table 3). This plant contains the most common mutant allele identified in the T1 generation (Fig. 4b), demonstrating stable transmission of this mutation. All of the remaining 22 T2 plants contained the FT-RGEN-B transgene. Among these plants, one possible *ft* homozygote was homozygous for the same allele found in FT-RGEN-B.2.10 (Table 3). A second possible *ft* homozygote was biallelic being heterozygous for the common one base pair insertion allele and for a 2 bp deletion allele (Table 3). These plants showed a strong late-flowering phenotype (Table 3). Additionally, the heterozygote candidate T2 plant for a 2 bp deletion allele was isolated (Fig. 5c). However, as these plants carry the FT-RGEN-B transgene, it is possible that the characterized mutant alleles were induced by additional rounds of mutagenesis in the T2 plants. The remaining T2 plants of this family that are not shown in Table 3 and had inherited the FT-RGEN-B T-DNA, did not carry the common mutant allele identified in the T1 plant. Nevertheless, many of these plants still exhibited late flowering of varying severity, as shown in Fig. 6a, suggesting new rounds of mutagenesis in the T2 generation. Consistently, sequence analysis using DNA extracted from these plants showed multiple peaks at the target site, implying a mixture of induced polymorphisms. To further analyze the inheritance of the induced mutations in FT-RGEN-B lineage, the genotypes of T3 progeny from FT-RGEN-B.2.7 were analyzed. Consistent with the results of FT-RGEN-A, independent segregation of the mutation detected in T2 plant with the RGEN transgene was observed in T3 population (Fig. 5d; Supplemental Table S4). Interestingly, we isolated the biallelic homozygous T3 plants which inherited the 2 bp deletion that was analyzed for inheritance and new candidates of heritable mutations (Supplemental Table S4), suggesting continuous generation of the diverse types of heritable mutations through the generation of RGEN transgenic plants. All of these plants also exhibited the late flowering phenotypes as shown in *ft* mutant.

**Table 3:** Transmission of induced polymorphisms to T2 progeny of FT-RGENs

Line number	Redetected polymorphism in T2 progeny	Estimated genotype of the redetected allele	FT-RGEN	Flowering time (Rosette leaf number)^a^
FT-RGEN-A.2.40	AGAGTGATTGATCTATTTAAA	Heterozygous	+	16
FT-RGEN-B.2.2	AAGCCAAGAGTTGAGATTTGG	Biallelic	+	31
	AAGCCAAGAGTTGAGA–GG			
FT-RGEN-B.2.3	AAGCCAAGAGTTGAGATTTGG	Heterozygous	+	22
FT-RGEN-B.2.6	AAGCCAAGAGTTGAGATTTGG	Heterozygous	+	28
FT-RGEN-B.2.7	AAGCCAAGAGTTGAGA–GG	Heterozygous	+	22
FT-RGEN-B.2.9	AAGCCAAGAGTTGAGATTTGG	Homozygous	+	25
FT-RGEN-B.2.10	AAGCCAAGAGTTGAGATTTGG	Heterozygous	−	13
FT-RGEN-B.2.18	AAGCCAAGAGTTGAGATTTGG	Homozygous	+	31
FT-RGEN-B.2.20	AAGCCAAGAGTTGAGATTTGG	Heterozygous	+	23
FT-RGEN-B.2.23	AAGCCAAGAGTTGAGATTTGG	Heterozygous	+	23
FT-RGEN-B.2.24	AAGCCAAGAGTTGAGATTTGG	Heterozygous	+	27

^a^Average flowering time of wild-type Col; 12.0 rosette leaves at bolting

Taken together, all of our results from FT-RGEN-A and FT-RGEN-B clearly demonstrate that the dividing tissue specific RGEN activity creates null mutations that are heritable, and that allelic diversity can be created by targeting the same gene with different *sgRNAs*.

## Discussion

**Fig. 7:**
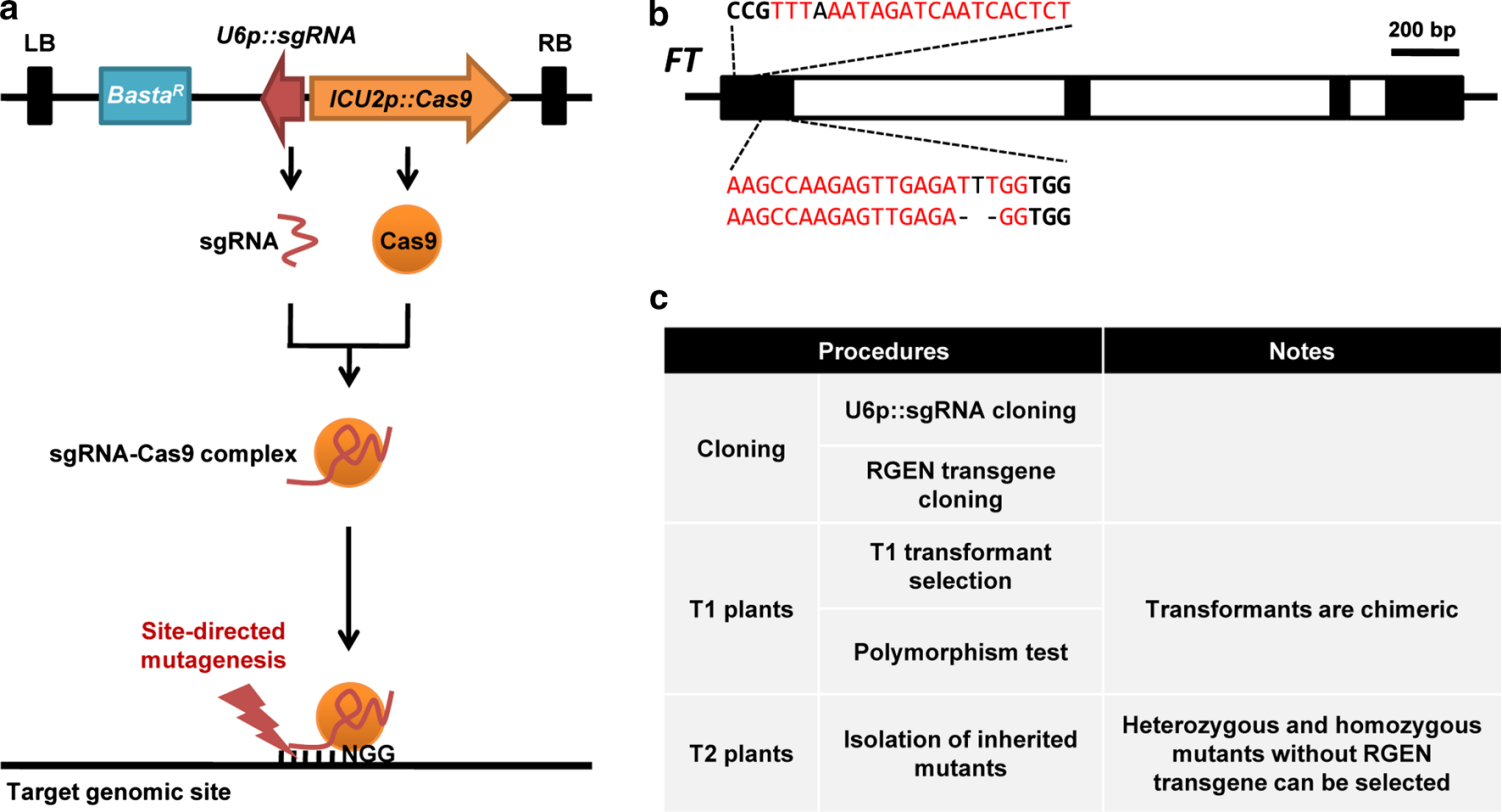
Schematic picture of RGEN system used in this study. **a** Schematic diagram of RGEN activity in plants. *Top* structure of T-DNA for RGEN is presented. Chimeric sgRNA and Cas9 are expressed from *U6p*::*sgRNA* and *ICU2p*::*Cas9* genes in the T-DNA inserted into the *Arabidopsis* genome. After RNA–protein complex formation, sgRNA guides the Cas9 to the target site by RNA–DNA complementary binding. On the *bottom*, DNA-bound sgRNA-Cas9 checks protospacer adjacent motif (PAM) for target validation and induces DNA cleavage. *LB* left border of T-DNA, *RB* right border of T-DNA, *Basta* ^*R*^ Basta resistance gene for selection of transgenic plants, *NGG* nucleotide sequences of PAM. **b** Established *ft* null mutant alleles recovered in this study. Genomic structure of *FT* locus is presented. *Black boxes* exons, *white boxes* introns, *scale bar* 200 bp. Nucleotide sequences of *sgRNA* target sites and NGG motifs are presented with *red* and *bold black letters*. Polymorphisms of newly isolated heritable *ft* mutant alleles in this study are marked with *black letters* within the target sequences. **c** Schematic procedure for the generation of mutant plants using RGEN system

In this study, we developed a single component transgenic system that facilitates RGEN-mediated mutagenesis in growing tissues including the meristematic region (Fig. 7a). We used this system to induce novel mutant alleles of *A. thaliana FT* that are predicted to abolish protein function by inducing frameshift mutations in two regions of the gene (Fig. 7b). Taken together, our results describe a powerful strategy to introduce transmissible mutations by the propagation of stable transgenic plants and we expect that this will be widely used for reverse genetics in *A. thaliana* (Fig. 7c).

The CRISPR/Cas system provides a robust method of gene targeting technology in diverse organisms, including higher plants. In contrast to previous reports in crop species (Li et al. 2013; Nekrasov et al. 2013; Shan et al. 2013), our study provides an alternative way to generate heritable site-directed mutations through the molecular breeding of transgenic plants that carry the RGEN components in their genomes (Fig. 7). As a result, we confirmed that the *ICU2p*::*Cas9/U6p*::*sgRNA* transgene modified two independent target sites of *FT* with high efficiency in the first generation of transformed plants (Figs. 3, 4). Remarkably, FT-RGEN-B plants showed the *ft* mutant late-flowering phenotype even in the T1 generation (Fig. 3d) and based on DNA sequencing of PCR products about 90 % of independent chromosomal DNA fragments were estimated to carry mutations at the target sites (Table 1). FT-RGEN-A and SPL4-RGEN transformants showed less modified target DNA than FT-RGEN-B plants (Table 1), suggesting a lower rate of mutagenesis by these RGENs. Differences in mutagenic efficiency of independent sgRNAs were reported also in other organisms (Cong et al. 2013; Hwang et al. 2013b; Li et al. 2013; Mali et al. 2013; Nekrasov et al. 2013; Shan et al. 2013). This variation in activity of sgRNA molecules might be due to differences in the chromatin state of target sites or/and the rate of sgRNA–Cas9 complex formation/guidance. However, the mutagenic efficiency of FT-RGEN-A and SPL4-RGEN was even higher than that of previous trials of RGENs in *A. thaliana* protoplasts (Li et al. 2013), and the FT-RGEN-A plant produced progeny that had inherited a mutant allele (Supplemental Table S3). This result implies that the RGEN approach in this study can bypass the technical limitations caused by inconsistent *sgRNA* activity for generation of transmissible mutations, although it is still crucial to understand why independent *sgRNAs* show variable activity in eukaryotic genomes. Recently, similar approaches were performed in *A. thaliana* using TALEN and RGEN techniques employing the *CaMV 35S* promoter, and those studies also isolated several transgenic plants carrying site-directed mutations (Christian et al. 2013; Feng et al. 2013). In combination with our study, these further support that RGEN transformant generation promises heritable genome engineering in higher plants as an alternative to mutant generation through regeneration by tissue culture.

To increase the transmission efficiency of RGEN-mediated polymorphisms, we aimed to cause mutation during embryogenesis using the *ICU2* promoter to express Cas9 (Fig. 1c). Mutation studies have demonstrated that mature *Arabidopsis* embryos contain on average two or three cells that give rise to the gametes, and mutation of either of these cells produces large mutant sectors leading to the production of many mutant progeny (Li and Redei 1969). Also cell lineages that give rise to vegetative sectors in mature plants can contribute to the axillary inflorescence reproductive tissue in *Arabidopsis* (Irish and Sussex 1992). In a recent study that presented heritable RGEN-mediated mutagenesis in *C. elegans* and zebra fish, the authors also used gonad germ line tissue and fertilized eggs for mutagenic induction to obtain inheritance, respectively (Cho et al. 2013b; Hwang et al. 2013a). We compared the frequency of detection of different alleles in T1 and T2 plants (Fig. 4). Notably a high rate of transmission to T2 progeny was observed in the case of the major polymorphism in the FT-RGEN-B T1.2 plant that was characterized in about 50 % of analyzed DNA (Table 3). This major polymorphism must have been induced early in development of the T1 plant, perhaps during embryogenesis. However, we could also observe the inheritance of additional mutant alleles that were found less frequently in T1 plants (Table 3). All of these aspects support the idea that mutagenesis by RGEN in growing tissues creates heritable genome modifications in *A. thaliana* and presumably other higher plants.

Variation in the occurrence of independent polymorphisms also suggests that particular types of polymorphisms are frequently induced at the cleavage site by the repair system in *A. thaliana*. We observed that a FT-RGEN-A T2 plant apparently heterozygous for an induced mutation produced T3 progeny that inherited the same mutant allele more frequently than expected for the progeny of a self-fertilized heterozygote (Supplemental Table S3). As the T2 plant contained the FT-RGEN-A transgene, one possibility is that the RGEN system independently induced the same mutant allele during the development of the T2 plant creating homozygous sectors. However, a second possibility is that homozygous sectors are generated by cleavage at the target site of the wild-type chromosome being repaired through homologous recombination with the mutant chromosome. Previously, it was reported using human cells that DNA double strand breaks (DSB) occurring during DNA replication are preferentially repaired by conservative homologous recombination (Saleh-Gohari and Helleday 2004). As we expressed Cas9 from the *ICU2* promoter that possesses the S-phase specific E2F *cis*-element (Barrero et al. 2007; Vandepoele et al. 2005), our RGEN system increases the likelihood that DNA cleavage is induced during DNA replication. If the nucleus in the replication process already contains one pair of mutant chromosomes and one pair of wild-type chromosomes, the RGEN would cleave only the target site in the wild-type chromosome. Repair of the DNA cleavage by homologous recombination would then use the mutant chromosome as a template creating homozygous sectors. Although it is necessary to examine whether this mechanism is conserved in plants, it suggests that the mutant allele induced in one pair of homologous chromosomes could be copied to another pair of wild-type homologous chromosomes in the same nucleus by our RGEN system and thereby increases the opportunity to obtain homozygous mutant progeny after self-fertilization of RGEN transformants.

In parallel with the recent advances in genome sequencing technologies, several new model plant species have been proposed to answer basic problems in plant biology. To delineate genetic mechanisms of interest in biological processes in such species, reverse genetics using the RGEN technique promises to be a powerful method. We employed the promoters of *U6 snRNA* and *ICU2* in *Arabidopsis* for induction of *sgRNA* and Cas9, respectively. *U6 snRNA* and *ICU2* encode factors for core cellular processes, mRNA splicing, and DNA replication (Barrero et al. 2007; Hyun et al. 2013; Li et al. 2007). Therefore, it is highly possible that the activity of promoters of *U6 snRNA* and *ICU2* are broadly conserved in higher plants. Consistent with this idea, the *U6 snRNA* promoter of *Arabidopsis* was used to induce *sgRNA* in tobacco protoplasts and it could result in site-directed mutation (Li et al. 2013). Plantlet regeneration from cultured callus is an alternative way of generating transgenic plants of many other species, including crops. We expect that our RGEN system can also be applied in these plant species for heritable site-directed mutagenesis because cell proliferation is crucial for callus formation and plantlet regeneration. The E2F S-phase specific *cis*-regulatory element is widely conserved in diverse organisms and is also present in the *ICU2* promoter that we used to drive *Cas9* expression (Vandepoele et al. 2005). Therefore our RGEN system may well also be powerful in inducing site-directed mutagenesis during cell proliferation of transformed callus and development of transgenic plantlets in a broad range of plant species. All of these aspects provide potential for our *U6p*::*sgRNA/ICU2p*::*Cas9* system to be employed in the creation of genetically modified material in diverse plant species, including those being established as new models.

## Electronic supplementary material

Below is the link to the electronic supplementary material.

Supplementary material 1 (DOCX 1229 kb)
