# Supplementary material for: Site-directed mutagenesis in Arabidopsis thaliana using dividing tissue-targeted RGEN of the CRISPR/Cas system to generate heritable null alleles
Source: Planta. 2014 Oct 1;241(1):271–84. doi: 10.1007/s00425-014-2180-5 (PMC4282705; doi:10.1007/s00425-014-2180-5)
Supplement: Supplementary file 1 — Supplementary material 1 (DOCX 1229 kb) [file 425_2014_2180_MOESM1_ESM.docx]

**SUPPLEMENTAL DATA**


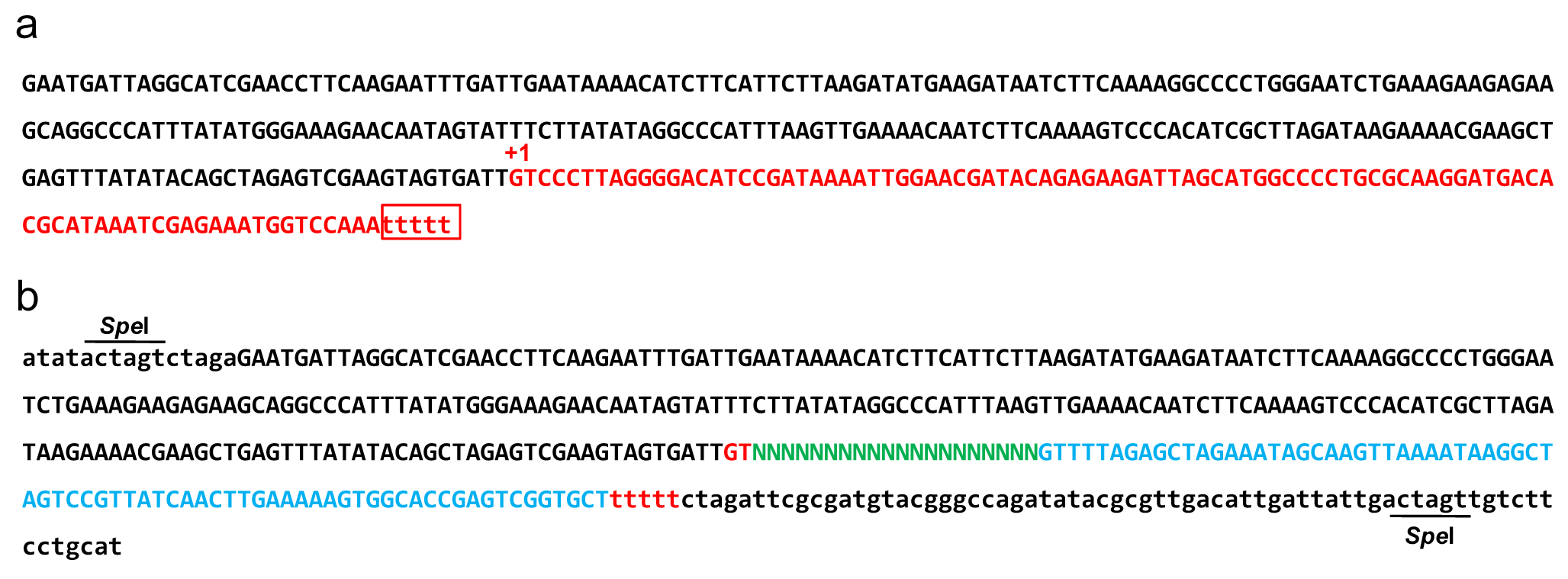


**Supplemental Fig. S1** Structures of *U6-26 snRNA* gene and *U6p::sgRNA* cassette.

**a** Nucleotide sequences of *U6-26 snRNA* gene in *Arabidopsis*. Sequences in black and red present promoter and transcribed regions of *U6 snRNA*, respectively. Transcriptional termination motif is marked with red boxes. **b** Nucleotide sequences of *U6p::sgRNA* cassette used in this study. Transcriptional region of *U6-26 snRNA* can be replaced with sgRNA by overlapping PCR (Fig. 2). Initial two nucleotides and terminator motif of *U6 snRNA* (marked with letters in red) are incorporated in the cassette for the proper initiation and termination of sgRNA transcription. Two *Spe*I restriction enzyme sites were added at both ends of the PCR products for the cloning of FT-RGENs.


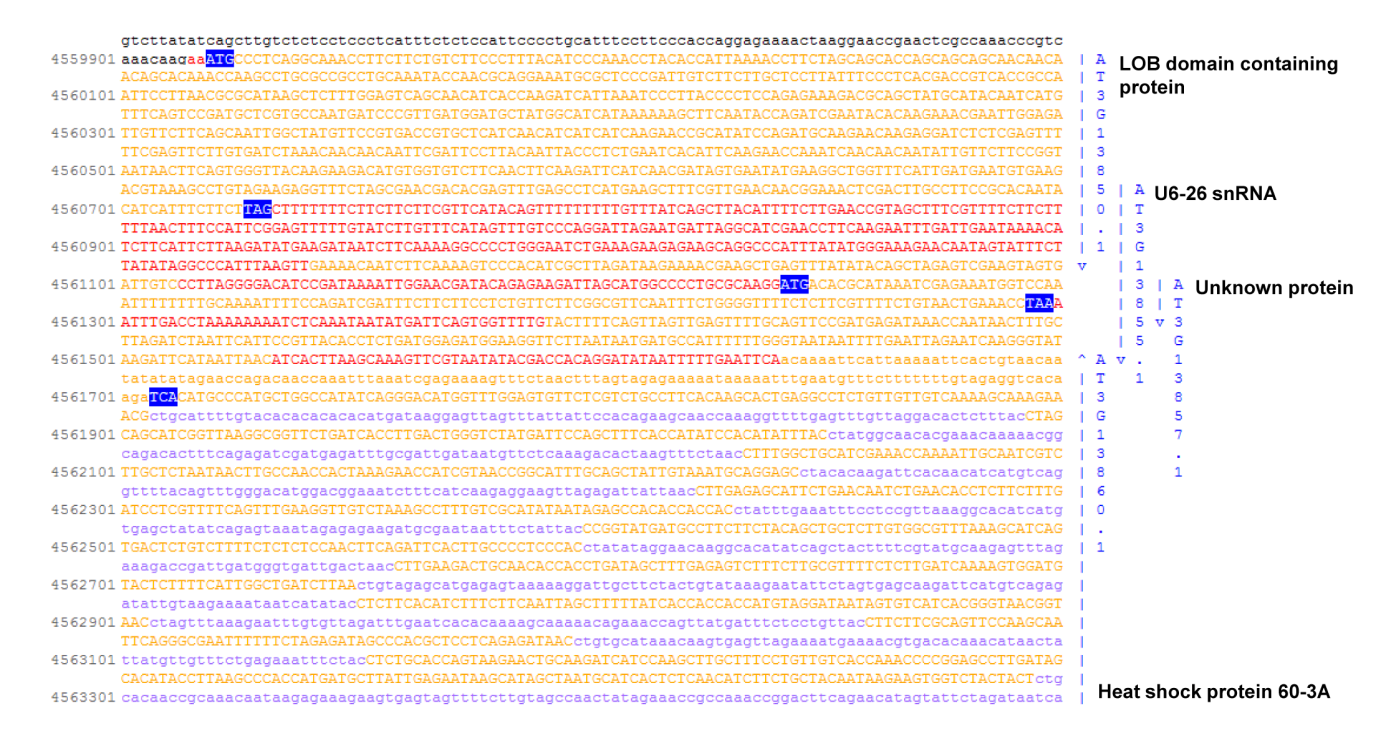


**Supplemental Fig. S2** Genomic structure of *U6-26 snRNA* region in *A. thaliana*.

The image was taken from Sequence Viewer tool on TAIR (www.arabidopsis.org) webpage. Nucleotide sequences in orange, purple and red represent exon, intron and UTR regions of genes in this region. Orientation and transcribed domain are marked with blue dotted-arrows on the right panel.


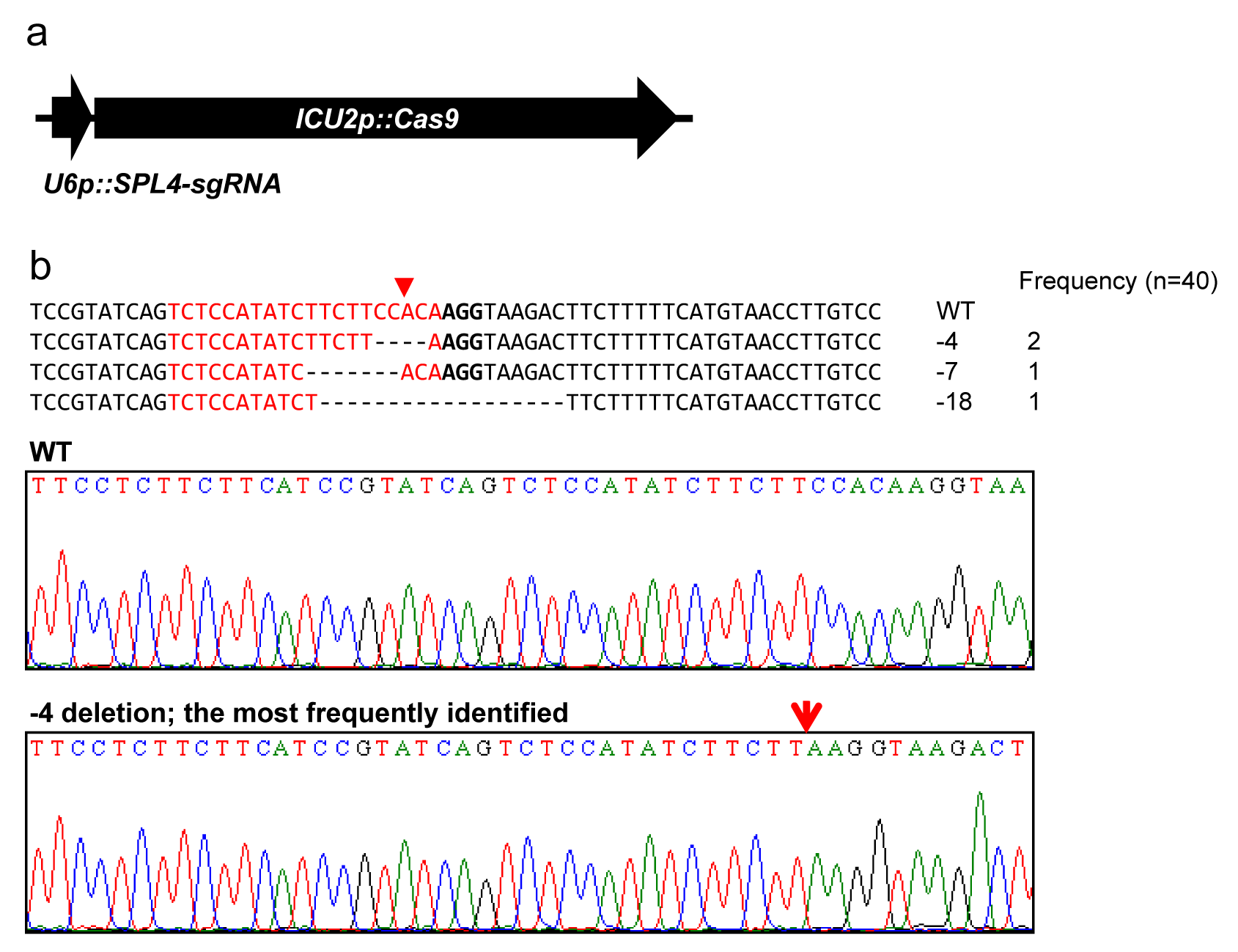


**Supplemental Fig. S3** Site-directed mutagenesis at *SPL4* using SPL4-RGEN.

**a** Schematic structure of SPL4-RGEN transgene. *U6p::sgRNA* which possesses a guide sequence for *SPL4* targeting was cloned upstream of *ICU2p::Cas9* in the same orientation. **b** Detected polymorphisms in SPL4-RGEN T1 plant. Representative polymorphisms are presented in the top panel. The predicted DNA cleavage site is marked with a red triangle. Nucleotide numbers of deletions and the frequency of each polymorphism are presented to the right of each polymorphism. Sequence peaks of wild-type and the most frequent polymorphism are presented at the bottom of the panel. The junction of nucleotide deletion at target site is marked with a red arrow.

**Supplemental Table S1** Flowering phenotypes of selected T1 transgenic plants of FT-RGENs

| Experiment 1 | | |
| --- | --- | --- |
| Genotype | Average rosette leaf number upon bolting  (number of analyzed plants) | |
| Col-0* | | 12.3 ± 0.8 (n=6) |
| *ft-10** | 38.2 ± 3.5 (n=3) | |
| FT-RGEN-A T1.1 | 10 | |
| FT-RGEN-A T1.2 | | 14 |
| FT-RGEN-A T1.3 | | 12 |
| FT-RGEN-A T1.4 | | 9 |
| FT-RGEN-A T1.5 | | 8 |
| FT-RGEN-A T1.6 | | 7 |
| FT-RGEN-A T1.7 | | 15 |
| FT-RGEN-A T1.8 | | 12 |
| FT-RGEN-A T1.9 | | 7 |
| FT-RGEN-A T1.10 | | 11 |
| FT-RGEN-A T1.11 | | 13 |
| FT-RGEN-A T1.12 | | 11 |
| FT-RGEN-B T1.1 | | 19 |
| FT-RGEN-B T1.2 | | 27 |
| Experiment 2 | | |
| Col-0* | | 11.0 ± 1.2 (n=6) |
| *ft-10** | | 32.0 ± 2.3 (n=6) |
| FT-RGEN-B T1.3 | | 13 |
| FT-RGEN-B T1.4 | | 20 |
| FT-RGEN-B T1.5 | | 17 |
| FT-RGEN-B T1.6 | | 9 |
| FT-RGEN-B T1.7 | | 15 |
| FT-RGEN-B T1.8 | | 25 |
| FT-RGEN-B T1.9 | | 19 |
| FT-RGEN-B T1.10 | | 16 |
| FT-RGEN-B T1.11 | | 18 |

*Col and *ft-10* were grown on soil from germination whereas the FT-RGEN transgenic plants were transferred to new soil after antibiotics selection.

**Supplemental Table S2** Characterized potential off-targets of FT-RGENs in *A. thaliana*.

| Off-target | Sequence | Chr. | Position | Strand | Number of mismatches |
| --- | --- | --- | --- | --- | --- |
| FT-RGEN-A | AGAGTGATTGATCTATTAAA**CGG** |  |  |  |  |
| off-target-1 | AGAGcGATTGAaCTATaAAA**CGG** | chr3 | 14974915 | + | 3 |
| off-target-2 | cGAGTGATgGATCTtTaAAA**CGG** | chr3 | 18233759 | + | 4 |
| off-target-3 | AGAGTGATTGATCgtTTcAt**CGG** | chr1 | 2388023 | - | 4 |
| off-target-4 | AGcaTGATTGATCTATcAAt**AGG** | chr1 | 6461769 | - | 4 |
| off-target-5 | AttGTGATTGATCaAaTAAA**TGG** | chr1 | 6609355 | - | 4 |
| off-target-6 | AGAGTGATTaATgTATTttA**AGG** | chr1 | 8990278 | + | 4 |
| off-target-7 | AGAGTGAgaGATCTcTTAAc**CGG** | chr1 | 10974637 | - | 4 |
| off-target-8 | AGAtgaATTGATaTATTAAA**AGG** | chr1 | 23081145 | + | 4 |
| off-target-9 | AaAtTGATctATCTATTAAA**AGG** | chr1 | 24678726 | + | 4 |
| off-target-10 | AaAGTGtTTtATCaATTAAA**GGG** | chr1 | 26470482 | - | 4 |
| off-target-11 | AGAcTaATTGATaTATTAAA**TGG** | chr1 | 27240505 | - | 3 |
| off-target-12 | AtttTGtTTGATCTATTAAA**AGG** | chr2 | 13493454 | - | 4 |
| off-target-13 | AGAGaGATTGgTaTATaAAA**AGG** | chr2 | 15972949 | + | 4 |
| off-target-14 | tGAGTGATTGtTCTAaaAAA**AGG** | chr5 | 2968118 | - | 4 |
| off-target-15 | AGAGTtAaTGcTCTATTgAA**TGG** | chr5 | 8115043 | + | 4 |
| off-target-16 | AaAGTGAagGATCaATTAAA**AGG** | chr5 | 9511927 | - | 4 |
| FT-RGEN-B | AAGCCAAGAGTTGAGATTGG**TGG** |  |  |  |  |
| off-target-1 | AAGCCAAGAGcTGAGgTgaG**AGG** | chr3 | 98423 | - | 4 |
| off-target-2 | AAGCaAAGAGcTaAGtTTGG**GGG** | chr1 | 16212607 | + | 4 |
| off-target-3 | AAGCaAAGAGcTaAGtTTGG**GGG** | chr1 | 16511399 | - | 4 |
| off-target-4 | cAGCCAAGAtcTcAGATTGG**TGG** | chr1 | 26841673 | - | 4 |
| off-target-5 | AAGCaAAGAGcTaAGtTTGG**GGG** | chr2 | 4957502 | + | 4 |
| off-target-6 | AAaCCcAGAGaTGAGtTTGG**AGG** | chr5 | 9926274 | - | 4 |
| off-target-7 | AAGCaAAGAGcTaAGtTTGG**GGG** | chr5 | 11765560 | - | 4 |
| off-target-8 | AAcCtgAGAGTTGAGcTTGG**AGG** | chr5 | 19292878 | - | 4 |
| off-target-9 | AAGCCtcGcGTTGAGATcGG**TGG** | chr5 | 24922959 | + | 4 |
| off-target-10 | AAGCaAAGAGcTaAGtTTGG**GGG** | chr4 | 4209597 | - | 4 |
| off-target-11 | AcGCaAAGAGTaaAGATTGG**TGG** | chr4 | 9587712 | + | 4 |
| off-target-12 | AAaCCAAtAGTgGAGATTGG**AGG** | chr4 | 11002792 | - | 3 |

**Supplemental Table S3** Transmission of the redetected allele in FT-RGEN-A.2.40 T2 plants to T3 progeny.

| T3 line | Flowering time* | Genotype of the redetected single T insertion | RGEN | T3 line | Flowering time* | Genotype of the redetected single T insertion | RGEN^+^ |
| --- | --- | --- | --- | --- | --- | --- | --- |
| A.2.40.2 | 32 | *ft/ft* | + | A.2.40.50 | 16 | *ft/+* | - |
| A.2.40.3 | 35 | *ft/ft* | + | A.2.40.51 | 35 | *ft/ft* | - |
| A.2.40.4 | 13 | wt | + | A.2.40.52 | 12 | wt | - |
| A.2.40.5 | 34 | *ft/ft* | - | A.2.40.53 | 12 | *ft/+* | - |
| A.2.40.6 | 15 | *ft/+* | - | A.2.40.54 | 10 | *ft/+* | + |
| A.2.40.7 | 33 | *ft/ft* | + | A.2.40.55 | 34 | *ft/ft* | - |
| A.2.40.10 | 13 | wt | - | A.2.40.56 | 17 | *ft/+* | + |
| A.2.40.12 | 13 | *ft/+* | - | A.2.40.57 | 27 | *ft/ft* | - |
| A.2.40.13 | 35 | *ft/ft* | + | A.2.40.58 | 17 | *ft/+* | + |
| A.2.40.14 | 12 | wt | - | A.2.40.59 | 34 | *ft/ft* | - |
| A.2.40.15 | 17 | *ft/+* | - | A.2.40.60 | 31 | *ft/ft* | + |
| A.2.40.16 | 35 | *ft/ft* | - | A.2.40.61 | 35 | *ft/ft* | + |
| A.2.40.17 | 12 | wt | - | A.2.40.62 | 11 | wt | - |
| A.2.40.18 | 14 | *ft/+* | - | A.2.40.63 | 33 | *ft/ft* | - |
| A.2.40.19 | 14 | *ft/+* | - | A.2.40.64 | 12 | wt | + |
| A.2.40.20 | 17 | *ft/+* | + | A.2.40.65 | 34 | *ft/ft* | - |
| A.2.40.21 | 15 | *ft/+* | - | A.2.40.66 | 13 | *ft/+* | + |
| A.2.40.22 | 32 | *ft/ft* | - | A.2.40.67 | 12 | *ft/+* | - |
| A.2.40.23 | 32 | *ft/ft* | - | A.2.40.68 | 33 | *ft/ft* | - |
| A.2.40.24 | 12 | wt | - | A.2.40.69 | 35 | *ft/ft* | + |
| A.2.40.25 | 17 | *ft/+* | + | A.2.40.70 | 32 | *ft/ft* | - |
| A.2.40.26 | 35 | *ft/ft* | - | A.2.40.71 | 11 | *ft/+* | - |
| A.2.40.27 | 32 | *ft/ft* | - | A.2.40.72 | 33 | *ft/ft* | - |
| A.2.40.28 | 18 | *ft/+* | + | A.2.40.73 | 11 | *ft/+* | - |
| A.2.40.29 | 33 | *ft/ft* | - | A.2.40.75 | 34 | *ft/ft* | + |
| A.2.40.30 | 12 | *ft/+* | - | A.2.40.76 | 36 | *ft/ft* | + |
| A.2.40.31 | 12 | wt | - | A.2.40.77 | 34 | *ft/ft* | - |
| A.2.40.32 | 32 | *ft/ft* | - | A.2.40.78 | 12 | wt | - |
| A.2.40.33 | 31 | *ft/ft* | - | A.2.40.79 | 34 | *ft/ft* | - |
| A.2.40.34 | 15 | *ft/+* | - | A.2.40.80 | 30 | *ft/ft* | - |
| A.2.40.35 | 15 | *ft/+* | - | A.2.40.81 | 32 | *ft/ft* | - |
| A.2.40.36 | 17 | *ft/+* | - | A.2.40.82 | 32 | *ft/ft* | - |
| A.2.40.37 | 33 | *ft/ft* | - | A.2.40.83 | 29 | *ft/ft* | - |
| A.2.40.38 | 13 | *ft/+* | - | A.2.40.84 | 15 | *ft/+* | - |
| A.2.40.39 | 32 | *ft/ft* | + | A.2.40.85 | 35 | *ft/ft* | - |
| A.2.40.40 | 15 | *ft/+* | - | A.2.40.86 | 13 | *ft/+* | - |
| A.2.40.41 | 14 | *ft/+* | + | A.2.40.87 | 10 | wt | + |
| A.2.40.42 | 36 | *ft/ft* | - | A.2.40.88 | 13 | *ft/+* | - |
| A.2.40.43 | 12 | wt | + | A.2.40.89 | 12 | *ft/+* | - |
| A.2.40.44 | 17 | *ft/+* | + | A.2.40.90 | 12 | *ft/+* | - |
| A.2.40.45 | 33 | *ft/ft* | + | A.2.40.91 | 12 | *ft/+* | + |
| A.2.40.46 | 11 | wt | + | A.2.40.92 | 13 | *ft/+* | - |
| A.2.40.47 | 19 | *ft/+* | + | A.2.40.93 | 14 | *ft/+* | + |
| A.2.40.48 | 36 | *ft/ft* | - | A.2.40.94 | 28 | *ft/ft* | - |
| A.2.40.49 | 34 | *ft/ft* | + | A.2.40.95 | 33 | *ft/ft* | - |

*Average flowering time of wild-type Col; 12.8 rosette leaves at bolting.

**Supplemental Table S4** Transmission of the redetected allele in FT-RGEN-B.2.7 T2 plants to T3 progeny.

| T3 line | Genotype of the redetected double T deletion | RGEN | Newly characterized heritable mutation | T3 line | Genotype of the redetected double T deletion | RGEN | Newly characterized heritable mutation |
| --- | --- | --- | --- | --- | --- | --- | --- |
| B.2.7.1 | *ft/+* | + |  | B.2.7.48 | *ft/+* | + |  |
| B.2.7.2 | wt | + |  | B.2.7.49 | *ft/+* | + |  |
| B.2.7.3 | *ft/ft* | - |  | B.2.7.50 | *ft/ft* | + |  |
| B.2.7.4 | *ft/+* | + |  | B.2.7.51 | *ft/ft* | + |  |
| B.2.7.5 | *ft/+* | + |  | B.2.7.54 | *ft/+* | + |  |
| B.2.7.6 | wt | + |  | B.2.7.55 | wt | + |  |
| B.2.7.7 | *ft/ft* | + |  | B.2.7.56 | *ft/+* | + |  |
| B.2.7.8 | wt | + |  | B.2.7.57 | *ft/+* | + |  |
| B.2.7.9 | *ft/+* | + |  | B.2.7.58 | *ft/ft* | - |  |
| B.2.7.10 | wt | + |  | B.2.7.59 | *ft/+* | + |  |
| B.2.7.11 | *ft/+* | - |  | B.2.7.60 | *ft/ft* | + |  |
| B.2.7.12 | *ft/+* | - |  | B.2.7.61 | wt | + |  |
| B.2.7.13 | *ft/+* | + |  | B.2.7.62 | wt | + |  |
| B.2.7.14 | wt | + |  | B.2.7.63 | *ft/+* | - |  |
| B.2.7.15 | *ft/+* | + |  | B.2.7.64 | *ft/+* | + | 25 bp deletion |
| B.2.7.16 | *ft/+* | + |  | B.2.7.65 | wt | - |  |
| B.2.7.17 | *ft/ft* | + |  | B.2.7.66 | wt | + |  |
| B.2.7.18 | wt | + |  | B.2.7.67 | *ft/ft* | + |  |
| B.2.7.19 | wt | + |  | B.2.7.68 | *ft/ft* | + |  |
| B.2.7.20 | *ft/+* | + |  | B.2.7.69 | *ft/+* | + |  |
| B.2.7.21 | *ft/+* | + | 9 bp deletion | B.2.7.70 | wt | + |  |
| B.2.7.22 | *ft/+* | + |  | B.2.7.71 | wt | + |  |
| B.2.7.23 | *ft/+* | - |  | B.2.7.72 | wt | + |  |
| B.2.7.24 | *ft/ft* | + |  | B.2.7.73 | wt | + | 1 bp T insertion homo |
| B.2.7.25 | *ft/ft* | + |  | B.2.7.74 | *ft/+* | - |  |
| B.2.7.27 | *ft/ft* | + |  | B.2.7.75 | *ft/+* | - |  |
| B.2.7.28 | *ft/ft* | + |  | B.2.7.76 | wt | + |  |
| B.2.7.29 | wt | - |  | B.2.7.77 | wt | + |  |
| B.2.7.31 | *ft/+* | + |  | B.2.7.78 | *ft/+* | + |  |
| B.2.7.32 | *ft/+* | + |  | B.2.7.79 | *ft/+* | - |  |
| B.2.7.33 | *ft/+* | + |  | B.2.7.80 | *ft/ft* | + |  |
| B.2.7.34 | *ft/ft* | + |  | B.2.7.81 | *ft/+* | + |  |
| B.2.7.36 | *ft/ft* | + |  | B.2.7.82 | *ft/+* | + |  |
| B.2.7.38 | *ft/+* | + |  | B.2.7.84 | *ft/+* | + | 1 bp T deletion |
| B.2.7.39 | *ft/+* | + |  | B.2.7.85 | wt | + |  |
| B.2.7.40 | *ft/+* | + |  | B.2.7.86 | *ft/ft* | + |  |
| B.2.7.41 | *ft/+* | - |  | B.2.7.87 | *ft/+* | + |  |
| B.2.7.42 | wt | + |  | B.2.7.88 | wt | - |  |
| B.2.7.43 | wt | - |  | B.2.7.89 | *ft/+* | + |  |
| B.2.7.44 | wt | + |  | B.2.7.90 | *ft/+* | + |  |
| B.2.7.45 | *ft/+* | + |  | B.2.7.93 | *ft/+* | + | nucleotide change |
| B.2.7.46 | *ft/+* | + |  | B.2.7.94 | *ft/ft* | + |  |
| B.2.7.47 | *ft/ft* | - |  | B.2.7.95 | *ft/+* | + |  |

**Supplemental Table S5** Sequences of primers used in this study.

| Primer | Sequence | Usage remark |
| --- | --- | --- |
| YH25-ICU2p-f | ATATACTAGTCAACGCTGCCAGATTCGATGT | Cloning of *ICU2* promoter |
| YH26-ICU2pCas9-r | GATGCTGTACTTCTTGTCCATTTTTACAAATCCGGTCAATTT |  |
| YH62-sg-1 | ATATACTAGTCTAGAGAATGATTAGGCATCGAACCT | Forward primer for *U6p::sgRNA* cassette |
| YH63-sg-2 | ATGCAGGAAGACAACTAGTCAA | Reverse primer for *U6p::sgRNA* cassette |
| YH64-FT-RGEN-A-1 | TTTAATAGATCAATCACTCTACAATCACTACTTCGACTCTAGCT | Reverse primer for *U6p* fragment with guide sequence |
| YH65-FT-RGEN-A-2 | AGAGTGATTGATCTATTAAAGTTTTAGAGCTAGAAATAGCAA | Forward primer for sgRNA fragment with guide sequence |
| YH66-FT-RGEN-B-1 | CCAATCTCAACTCTTGGCTTACAATCACTACTTCGACTCTAGCT | Reverse primer for *U6p* fragment with guide sequence |
| YH67-FT-RGEN-B-2 | AAGCCAAGAGTTGAGATTGGGTTTTAGAGCTAGAAATAGCAA | Forward primer for sgRNA fragment with guide sequence |
| YH92-FT-T7E1-f | CAACACAGAGAAACCACCTGT | T7E1 assay |
| YH93-FT-T7E1-r1 | CATCTGGATCCACCATAACCT |  |
| YH94-FT-T7E1-r2 | AACCAAGGTCTCTGCATGCC |  |
